# Assessing quality of life in patients with prostate cancer: a systematic and standardized comparison of available instruments

**DOI:** 10.1007/s11136-014-0678-8

**Published:** 2014-04-19

**Authors:** Stefanie Schmidt, Olatz Garin, Yolanda Pardo, José M. Valderas, Jordi Alonso, Pablo Rebollo, Luis Rajmil, Carlos Garcia-Forero, Montse Ferrer, the EMPRO Group

**Affiliations:** 1 Health Services Research Group, IMIM (Hospital del Mar Medical Research Institute), Doctor Aiguader 88, 08003 Barcelona, Spain; 2 Department of Experimental and Health Sciences, Pompeu Fabra University (UPF), Doctor Aiguader 88, Barcelona, Spain; 3 Biomedical Research Center Network Group, Epidemiology and Public Health (CIBERESP), Instituto de Salud Carlos III, Melchor Fernández Almagro 3-5, 28029 Madrid, Spain; 4 Health Services and Policy Research Group, University of Exeter Medical School, University of Exeter, St Luke’s Campus, Smeall Building, Exeter, EX1 2LU UK; 5 BAP Health Outcomes, LA-SER Group, C/ Azcárraga 12a, 33010 Oviedo, Spain; 6 Catalan Agency for Health Information, Assessment and Quality (CAHIAQ), Pl. Lesseps 1, Barcelona, Spain; 7 Universitat Autònoma de Barcelona (UAB), Plaça Cívica, 08193 Cerdanyola del Vallès, Barcelona Spain

**Keywords:** Prostatic neoplasms, Quality of life, Patient outcomes, Psychometrics, Validation studies

## Abstract

**Purpose:**

The objective was to obtain a standardized evaluation of available prostate cancer-specific quality of life instruments used in patients with early-stage disease.

**Methods:**

We carried out systematic literature reviews in the PubMed database to identify manuscripts which contained information regarding either the development process or metric properties of prostate cancer-specific quality of life instruments. Each instrument was evaluated by two experts, independently, using the Evaluating Measures of Patient-Reported Outcomes (EMPRO) tool. An overall and seven attribute-specific EMPRO scores were calculated (range 0–100, worst to best): measurement model, reliability, validity, responsiveness, interpretability, burden and alternative forms.

**Results:**

Eight instruments and 57 manuscripts (2–15 per instrument) were identified. The Expanded Prostate Cancer Index Composite (EPIC) was the best rated (overall EMPRO score 83.1 points). Good results were also obtained by University of California Los Angeles-Prostate Cancer Index (UCLA-PCI), Patient-Oriented Prostate Utility Scale (PORPUS) and Prostate Cancer Quality of Life Instrument (PC-QoL) with 77.3, 70.5 and 64.8 points, respectively. These four instruments passed with distinction the validity and responsiveness evaluation. Insufficient reliability results were observed for UCLA-PCI and PORPUS.

**Conclusions:**

Current evidence supports the choice of EPIC, PORPUS or PC-QoL. Attribute-specific EMPRO results facilitate selecting the adequate instrument for every purpose. For longitudinal studies or clinical trials, where responsiveness is the priority, EPIC or PC-QoL should be considered. We recommend the PORPUS for economic evaluations because it allows cost-utility analysis, and EPIC short versions to minimize administration burden.

**Electronic supplementary material:**

The online version of this article (doi:10.1007/s11136-014-0678-8) contains supplementary material, which is available to authorized users.

## Introduction

Prostate cancer is currently the most frequent solid neoplasm and the third cause of death in European men [1]. The increased tumor detection is associated with the use of the prostate-specific antigen testing, which changed the epidemiology of this tumor, by moving diagnosis to younger patients at earlier stages. Now, men have to live longer with their disease and with the treatment’s side effects, which are mainly urinary, sexual and bowel problems [2, 3]. Therefore, Patient-Reported Outcomes (PROs), such as health-related quality of life (HRQL), have achieved an important role in the evaluation of treatment benefits and harms in these patients [4, 5]. The first prostate cancer-specific HRQL instruments, such as the prostate module of the European Organization for Research and Treatment of Cancer (EORTC QLM-P14) [6] or the Prostate Cancer-Specific Quality of Life Instrument (PROSQOLI) [7], were designed mainly for patients in advanced disease stages and present significant limitations when used in patients with localized disease.

The need for tools capable of capturing all relevant aspects in patients diagnosed at early stages of disease led to the development of several prostate cancer-specific instruments. A recent systematic review [8] identified almost 30 symptom measures either designed or adapted for prostate cancer patients. Several share a similar content and applicability, which makes it a complicated task to select the right instrument for a specific purpose and setting, calling for the need to evaluate those measures considering their strengths and weaknesses. The right choice depends on both the instrument’s characteristics and the specific study requirements (mainly objectives and available resources). A comparative evaluation among instruments would be of great value to facilitate this selection task.

Several attempts have been made to systemize evaluation criteria for PROs. The GraQol Index was the first instrument that generated a global score [9]. Currently, there are two other tools used for this purpose, the COnsensus-based Standards for the selection of health status Measurement INstruments (COSMIN) [10], and the Evaluating Measures of Patient-Reported Outcomes (EMPRO) [11]. While the COSMIN was developed as a checklist for evaluating the methodological quality of each individual study, the EMPRO was designed to assess the quality of the PRO measure by taking into account all the available studies. EMPRO considers both the methods applied in the studies and the adequacy of the results.

The quality of a PRO measure was defined by the EMPRO developers as the “degree of confidence that all possible bias has been minimized and that the information about the process which led to its development and evaluation is clear and accessible” [11]. The EMPRO combines 3 fundamental aspects: (1) well-described and established attributes for assessment, (2) expert reviewers to conduct the assessment, and (3) scores that allow a direct comparison among outcome measures. It is based on an exhaustive series of recommendations regarding the ideal attributes of PRO measures [12]. The EMPRO is a valid and reliable tool that has proven its usefulness in comparing the performance of generic [11] and disease-specific PROs, such as heart failure [13] and shoulder disorders [14].

Reviews have been published which identify [15], classify [16–20] or evaluate [8, 21, 22] PRO measures for prostate cancer patients. However, none of these reviews used a validated tool for the evaluation. The focus of the latter three evaluative reviews differed a lot: from generic, cancer- and prostate cancer-specific PRO instruments [21, 22] to symptom measures [8]. The number of instruments evaluated varied accordingly from 16 [22] to 29 [8]. Our study focus was set on instruments measuring the impact of localized prostate cancer and treatment side effects on patients’ HRQL, and not just measuring the frequency of symptoms. The aim of our study was to obtain a systematic and standardized EMPRO evaluation of the evidence available on development process, metric properties and administration issues of prostate cancer-specific HRQL instruments that are currently applicable in patients with early-stage disease.

## Methods

### Systematic review

We identified the prostate cancer-specific HRQL instruments by reviewing the Patient-Reported Outcomes and Quality of Life Instruments Database (PROQOLID) [23] and the websites of two cancer research groups: European Organization for Research and Treatment of Cancer (EORTC)^1^ and Functional Assessment of Cancer Therapy Group (FACT).^2^ We also examined topic-related review articles [8, 15–22] and their bibliographic reference lists. We included prostate cancer-specific HRQL instruments that were applicable to patients with localized disease. We excluded instruments that are domain- or treatment-specific, such as the Sexual Health Inventory for Men instrument [24], or the Prostatectomy Therapy Survey Instrument [25].

Once the instruments were identified (five through PROQOLID, EORTC and FACT; and three through review articles in PubMed), we carried out systematic searches for each instrument in the PubMed database (September 2013) in order to obtain all the available published evidence. The search strategy combined the keywords “urologic cancer” or “prostate cancer” and “quality of life” and the name of the instrument (full name and abbreviation), both as MeSH terms and free-text entries (see Online Appendix 1). Articles were eligible for inclusion if they contained information regarding the development process of the instrument, its metric properties and administration issues. We only considered original research articles published in English, Spanish, French or German.

In a two-step process, abstracts and full-text articles were independently reviewed by two investigators (S.S. and Virginia Becerra). A third investigator (M.F.) mediated and resolved discrepancies in each step. We then manually examined the bibliographic reference lists of the articles selected for full review.

### Evaluating Measures of Patient-Reported Outcomes (EMPRO)

The EMPRO [11] was designed to measure the quality of PRO instruments. It assesses quality as an overall concept, which is based on eight attributes (39 items) covering: “conceptual and measurement model” (concepts and population intended to assess); “reliability” (to which degree an instrument is free of random error); “validity” (to which degree an instrument measures what it intends); “responsiveness” (ability to detect change over time); “interpretability” (assignment of meanings to instruments’ scores); “burden” (time, effort and other demands for administration and response); “alternative modes of administration” (i.e., self- or interviewer-administered, telephone or computer-assisted interview); and “cross-cultural and linguistic adaptations” (equivalence across translated versions). For instruments which had some country versions available (e.g., Canadian, Dutch, Italian, Japanese and Spanish [26–30] University of California Los Angeles-Prostate Cancer Index (UCLA-PCI) versions), their studies were considered in the EMPRO evaluation. Nevertheless, the “cross-cultural and linguistic adaptation” attribute was not completed because the separate evaluation of every version was beyond the scope of this study.

All EMPRO attributes and items are accompanied by a short description to facilitate understanding the intended meaning and to guarantee a standardized application during the evaluation process. The item content for each attribute is summarized in the table of EMPRO results. Agreement with each item can be answered on a four-point Likert’s scale, from 4 (strongly agree) to 1 (strongly disagree). The “no information” box can be checked in case of insufficient information. Five items allow replying with “not applicable.” It is recommended to provide detailed comments to justify each EMPRO rating. These comments aid in the interpretation of the EMPRO scores.

### Standardized EMPRO evaluation

Each prostate cancer-specific instrument was evaluated by two different experts using the EMPRO tool. Experts were identified and invited because of their expertise and experience in PRO measurement: Eight were senior researchers who belonged to the EMPRO tool development working group, and the other eight were junior researchers who had previously been certified as EMPRO experts after participating in a training course and successfully completing a supervised evaluation. The review pairs were composed of one senior and one junior researcher. In order to minimize the potential bias, experts were not authors nor had been involved in the development or adaptation process of their assigned instrument.

The EMPRO evaluation process consisted of two consecutive rounds. In the first round, every expert independently evaluated his or her assigned instrument by reviewing the full-text articles identified through the systematic review process and by applying the EMPRO tool [11]. In the second round, each expert was provided with the rating results of the other expert who had this instrument assigned. In case of discrepancies, first, they were invited to resolve them through consensus, and second, if necessary, they were solved by a third reviewer.

### Statistical analysis

Attribute-specific scores and an overall score were calculated. Detailed information and algorithms to obtain EMPRO scores are available online.^3^ First, the mean of the applicable items was calculated for each attribute (when at least 50 % of them were rated); and second, this raw mean was linearly transformed into a range of 0 (worst possible score)–100 (best possible score). Items for which the response option “no information” had been selected were assigned a score of 1 (lowest possible score). Separate subscores for the “reliability” and “burden” attributes were calculated as they are composed of two components each: “internal consistency” and “reproducibility” for reliability, as well as “respondent” and “administrative” for burden. For reliability, the highest subscore for the two components was then chosen to represent the attribute.

Besides the attribute-specific scores, an overall score was computed by calculating the mean of the five metric-related attributes: “conceptual and measurement model,” “reliability,” “validity,” “responsiveness to change” and “interpretability.” The overall score was only calculated when at least three of these five attributes had a score. EMPRO scores were considered reasonably acceptable if they reached at least 50 points (out of the 100 maximum theoretical points). This threshold was chosen based on the global recommendations made by the reviewers in the first two EMPRO studies [11, 13]. The receiver operating characteristic (ROC) curve was calculated to evaluate the agreement between EMPRO attribute scores and the reviewers’ global recommendations. The area under the ROC curve was of 0.87 and the suggested cutoff was 51 (data not shown but available upon request).

## Results

### Characteristics of instruments

We identified eight HRQL instruments applicable to patients with early-stage prostate cancer, which were developed between 1997 and 2008 (Table 1). Four instruments were designed for all tumor stages (Estudio sobre la Calidad de Vida en el Cáncer de Próstata—ESCAP-CDV [31], EORTC QLQ-PR25 [32], FACT-P [33], and Patient-Oriented Prostate Utility Scale—PORPUS [34]) and the other four were developed specifically for patients at early-stage disease (Expanded Prostate Cancer Index Composite—EPIC [35], Prostate Cancer Quality of Life Instrument—PC-QoL [36], Prostate Cancer Symptom Indices—PCSI [37] and UCLA-PCI [38]). The EORTC QLQ-PR25 [32] and FACT-P [33] are tumor location-specific modules and were developed to complement the corresponding cancer-specific core questionnaire that measures general well-being (EORTC QLQ-C30 and FACT-General, respectively). The ESCAP-CDV [31] is a Spanish instrument which covers eight dimensions of general health and one prostate cancer-specific module. The PORPUS [34] is a unidimensional utility instrument composed by five general health and five prostate cancer-specific questions. Most of the instruments differentiate among bowel, sexual and urinary domains. EPIC [35] was developed from the UCLA-PCI [38] by supplementing it with items focusing on urinary irritative and obstructive voiding symptoms, as well as a hormonal domain. EORTC-PR25 and EPIC are the only instruments that consider the whole symptom spectrum (urinary, bowel, sexual and hormonal) in their content.

**Table 1:** Summarized characteristics of the evaluated prostate cancer-specific quality of life instruments

Instrument	Author	No. of manuscripts^a^	Purpose of development	Disease stage	Response option; score range	Time framework	No. of items (time to complete)	No. of domains	Domains measured (no. of items)			
									Bowel	Sexual	Urinary	Other
1. ESCAP-CDV	Morales et al. [31]	2	To design a prostate cancer-specific QoL instrument valid in Spanish population, based on the EORTC QLQ-C3	All stages	Four-point Likert’s (lower scores mean better QoL)	Last 4 weeks	34 (10′)	9	–	–	–	Eight general health domains (30) One prostate cancer-specific domain (6)
2. EORTC QLQ-PR25	Van Andel et al. [32]	5 (3)	To assess treatment-related complications of prostate cancer therapy	All stages	Four-point Likert’s; 0–100^b^	Last 1–4 weeks	25 (15′)	6	B. symptom (4)	S. active (2) S. functioning (1^c^)	U. symptom (8) Incontinence aid (1^c^)	Hormonal symptom (6)
3. EPIC	Wei et al. [35]	13 (4)	To facilitate a more comprehensive QoL assessment by capturing impact of new treatments	Early stage	Five-point Likert’s; 0–100 (worst to best)	Last 4 weeks	50 (20′ with SF-12)	4 (8 subscales)	B. summary (14) B. function (7) B. bother (7)	S. summary (13) S. function (9) S. bother (4)	U. summary (12) U. incontinence (4) U. irritative-obstructive (7)	Hormonal summary (11) Hormonal function (5) Hormonal bother (6)
4. FACT-P	Esper et al. [33]	12 (3)	To assess treatment-related complications of prostate cancer therapy	All stages	Five-point Likert’s; 0–48 (worst to best)	Last week	12 (8–10′)	1	–	–	–	–
5. PC-QoL	Giesler et al. [36]	3	To develop a comprehensive instrument for use in clinic and research settings	Early stage	4–6-point Likert’s; 0–100 (worst to best)	Last 4 weeks	52 (15′)	10	B. function (7) B. role activity limitation (5) B. bother (4)	S. function (7) S. role activity limitation (5) S. bother (6)	U. function (5) U. role activity limitation (5) U. bother (4)	Cancer worry (4)
6. PCSI	Clark et al. [37]	5	To assess treatment-related complications of early prostate cancer therapy	Early Stage	4–5-point Likert’s; 0–100 (best to worst)	Last 1–4 weeks	29 (n.i.)	8	B. dysfunction (6) B. symptom distress (4)	S. dysfunction (5) S. symptom distress (2)	Incontinence dysfunction (3) O–I dysfunction (5) Incontinence distress (1) O–I distress (5)	–
7. PORPUS	Krahn et al. [34]	5	To develop a health-state classification system for multiple purposes (econometric and psychometric methods)	All stages	4–6-point Likert’s; 0–1 (death to best health)	Last 2 weeks	10 (n.i.)	1	–	–	–	–
8. UCLA-PCI	Litwin et al. [38]	16 (5)	To assess health concerns central to patients that undergo surgery or radiotherapy	Early stage	3–5-point Likert’s; 0–100 (worst to best)	Last 4 weeks	20 (20′ with SF-36)	6	B. function (4) B. bother (1)	S. function (8) S. bother (1)	U. function (5) U. bother (1)	–

Instruments: *ESCAP*-*CDV* Estudio sobre la Calidad de Vida en el Cáncer de Próstata, *EORTC QLQ*-*PR25* European Organization for Research and Treatment in Cancer, Quality of Life Group-Prostate Cancer Module, *EPIC* Expanded Prostate Cancer Index Composite, *FACT*-*P* Functional Assessment of Cancer Therapy-Prostate Cancer Module, *PC*-*QoL* Prostate Cancer Quality of Life Instrument, *PCSI* Prostate Cancer Symptom Indices, *PORPUS* Patient-Oriented Prostate Utility Scale, *UCLA*-*PCI* University of California Los Angeles-Prostate Cancer Index

*O*–*I* obstruction/irritation, *n.i.* no information, *QoL* quality of life

^a^Number of manuscripts used in the EMPRO evaluation. In brackets, the number of manuscripts reporting studies performed with country-specific versions

^b^Higher scores reflecting either more symptoms (urinary, bowel, hormonal) or higher levels of functioning (sexual)

^c^Conditional item

### Retrieved information

The number of articles initially retrieved from the systematic literature search varied a lot, ranging from 323 (UCLA-PCI) to only two (ESCAP-CDV). The results of the systematic review process are described in Table 2. Most of the articles were excluded because they were not related to the instrument or did not provide any information on development process, metric properties or administration issues. The final number of articles included in the EMPRO evaluation varied from 16 (UCLA-PCI) to two (ESCAP-CDV) (Table 1). The bibliographic references of the included studies are shown in the Online Appendix 2.

**Table 2:** Results of the systematic literature review. Number of manuscripts identified, excluded and used in the EMPRO evaluation

Instrument: abbreviation and full name	Total manuscripts identified	Manuscripts excluded				Manuscripts with metric information (country-specific)
		Without instrument information	Without metric information	Other language	Total excluded	
ESCAP-CDV	2	–	–	–	0	2
EORTC QLQ-PR25	236	181	51	–	232	5 (3)
EPIC	236	70	151	2	223	13 (4)
FACT-P	182	109	59	2	170	12 (3)
PC-QoL	145	132	10	–	142	3
PCSI	27	15	7	–	22	5
PORPUS	12	2	6	–	8	5
UCLA-PCI	323	91	216	1	307	16 (5)

Instruments: *ESCAP-CDV* Estudio sobre la Calidad de Vida en el Cáncer de Próstata, *EORTC QLQ-PR25* European Organization for Research and Treatment in Cancer, Quality of Life Group-Prostate Cancer Module, *EPIC* Expanded Prostate Cancer Index Composite, *FACT-P* Functional Assessment of Cancer Therapy-Prostate Cancer Module, *PC-QoL* Prostate Cancer Quality of Life Instrument, *PCSI* Prostate Cancer Symptom Indices, *PORPUS* Patient-Oriented Prostate Utility Scale, *UCLA-PCI* University of California Los Angeles-Prostate Cancer Index

### Results of the EMPRO ratings

**Fig. 1:**
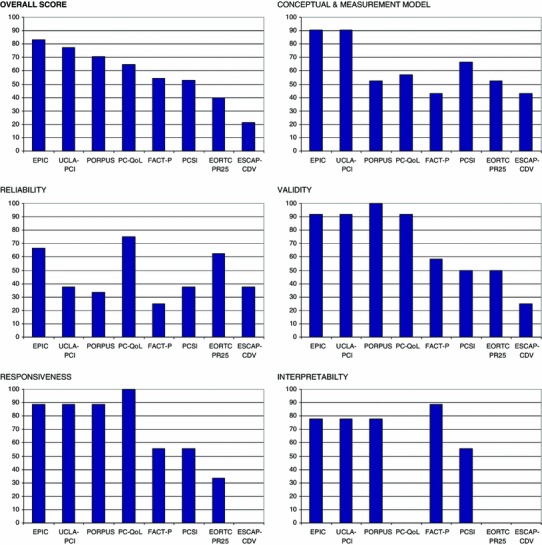
Overall ranking of instruments and their attribute-specific EMPRO scores. EMPRO scores ranged 0–100 (worst to best). Instruments: *ESCAP*-*CDV* Estudio sobre la Calidad de Vida en el Cáncer de Próstata, *EORTC QLQ*-*PR25* European Organization for Research and Treatment in Cancer, Quality of Life Group-Prostate Cancer Module, *EPIC* Expanded Prostate Cancer Index Composite, *FACT*-*P* Functional Assessment of Cancer Therapy-Prostate Cancer Module, *PC*-*QoL* Prostate Cancer Quality of Life Instrument, *PCSI* Prostate Cancer Symptom Indices, *PORPUS* Patient-Oriented Prostate Utility Scale, *UCLA*-*PCI* University of California Los Angeles-Prostate Cancer Index

Detailed EMPRO results of the standardized evaluation are presented in Table 3 and summarized in figure 1. Consensus between the two experts of an instrument was achieved in almost all cases, and the third expert was only needed to solve discrepancies for one instrument. The overall score, which summarizes the five attribute-specific scores described above, ranged from 83.1 (EPIC) to 21.1 (ESCAP-CDV). In the “conceptual and measurement model” attribute, instruments scored from 90.5 (EPIC, UCLA-PCI) to 42.9 (ESCAP-CDV, FACT-P), with six out of eight instruments presenting scores higher than 50. “Reliability” scores ranged from 75 (PC-QoL) to 25 (FACT-P), and only three instruments scored above the threshold of 50. “Validity” scores ranged from 100 (PORPUS) to 25.0, with only one instrument below 50 (ESCAP-CDV). In “responsiveness,” instruments scored from 100 (PC-QoL) to 33.3 (EORTC-PR25), and six out of eight instruments scored higher than 50. “Interpretability” scores were highest for FACT-P (88.9), followed by EPIC, PORPUS and UCLA-PCI (each 77.8), though no information was found for three instruments. UCLA-PCI and PC-QOL presented the lowest respondent burden (66.7 and 55.6 points, respectively) and, together with EPIC, also the lowest administrative burden (ranging from 91.7 to 75 points).

**Table 3:** Ratings of each EMPRO item and attribute for every prostate cancer-specific quality of life instrument identified

Attributes	ESCAP-CDV	EORTC PR25	EPIC	FACT-P	PC-QoL	PCSI	PORPUS	UCLA-PCI
Concept and measurement model	42.9	52.4	90.5	42.9	57.1	66.7	52.4	90.5
1. Concept of measurement stated	++++	++++	++++	++++	++++	++++	++++	++++
2. Obtaining and combining items described	++	++	++++	++	+++	++++	++++	++++
3. Rationality for dimensionality and scales	++	++	++++	+	+++	++++	++	++++
4. Involvement of target population	++	+++	++++	+++	++++	+++	++++	++++
5. Scale variability described and adequate	++	++++	+++	++	+++	++	++	++++
6. Level of measurement described	++	+	+++	+	–	++	+	++
7. Procedures for deriving scores	++	++	++++	+++	+	++	+	++++
Reliability—total score	37.5	62.5	66.7	25.0	75	37.5	33.3	37.5
Reliability: internal consistency	37.5	62.5	62.5	25.0	75	37.5		37.5
8. Data collection methods described	+++	++++	++++	++	++++	+++	–	++
9. Cronbach’s alpha adequate	++	+++	+++	++	++++	++	–	+++
10. IRT estimates provided	–	–	–	–	–	–	–	–
11. Testing in different populations	n.a.	n.a.	n.a.	n.a.	n.a.	n.a.	n.a.	n.a.
Reliability: reproducibility	33.3		66.7	0	50	16.7	33.3	33.3
12. Data collection methods described	++	–	+++	+	++++	++	+++	+++
13. Test–retest and time interval adequate	++	–	++++	+	+++	++	++	++
14. Reproducibility coefficients adequate	+++	–	++++	+	++	–	++	++
15. IRT estimates provided	–	+++	–	–	–	–	–	–
Validity	25.0	50	91.7	58.3	91.7	50	100	91.7
16. Content validity adequate	++	+	+++	+++	+++	++	++++	++++
17. Construct/criterion validity adequate	++	++++	++++	+++	++++	++	++++	+++
18. Sample composition described	+	+++	++++	++	++++	+++	++++	++++
19. Prior hypothesis stated	++	++	++++	+++	++++	+++	++++	++++
20. Rational for criterion validity	n.a.	n.a.	n.a.	n.a.	n.a.	n.a.	n.a.	n.a.
21. Tested in different populations	n.a.	n.a.	n.a.	n.a.	n.a.	n.a.	n.a.	n.a.
Responsiveness		33.3	88.9	55.6	100	55.6	88.9	88.9
22. Adequacy of methods	–	+++	++++	+++	++++	+++	+++	+++
23. Description of estimated magnitude of change	–	++	++++	+++	++++	+++	++++	++++
24. Comparison of stable and unstable groups	–	–	+++	++	++++	++	++++	++++
Interpretability			77.8	88.9		55.6	77.8	77.8
25. Rational of external criteria	–	–	+++	+++	–	+++	++++	+++
26. Description of interpretation strategies	–	–	+++	++++	–	+++	++	+++
27. How data should be reported stated	–	–	++++	++++	–	++	++++	++++
**OVERALL SCORE**	**21.1**	**39.7**	**83.1**	**54.1**	**64.8**	**53.1**	**70.5**	**77.3**
Burden								
Burden: respondent	22.2	33.3	44.4	22.2	55.6		0	66.7
28. Skills and time needed	++	++	++	++	++++	–	+	++++
29. Impact on respondents	++	+++	++++	++	+++	–	+	++++
30. Not suitable circumstances	–	–	–	–	–	–	–	–
Burden: administrative			91.7		75		8.3	91.7
31. Resources required	–	–	++++	–	++++	–	+	++++
32. Time required	–	–	++++	–	++++	–	++	++++
33. Training and expertise needed	–	–	+++	–	++++	–	–	++++
34. Burden of score calculation	++	+	++++	+	–	–	–	+++

Explanation: ++++ 4 (strongly agree), +++ 3, ++ 2, + 1 (strongly disagree), – no information, *n.a.* not applicable. The higher the agreement the better the rating

Instruments: *ESCAP*-*CDV* Estudio sobre la Calidad de Vida en el Cáncer de Próstata, *EORTC QLQ*-*PR25* European Organization for Research and Treatment in Cancer, Quality of Life Group-Prostate Cancer Module, *EPIC* Expanded Prostate Cancer Index Composite, *FACT*-*P* Functional Assessment of Cancer Therapy-Prostate Cancer Module, *PC*-*QoL* Prostate Cancer Quality of Life Instrument, *PCSI* Prostate Cancer Symptom Indices, *PORPUS* Patient-Oriented Prostate Utility Scale, *UCLA*-*PCI* University of California Los Angeles-Prostate Cancer Index

EPIC and UCLA-PCI provide alternative forms of administration, as well as short forms whose evaluation is shown in Table 4. Apart from the traditional paper mode, there is a web administration form for UCLA-PCI [39] and a telephone administration with interactive voice response for EPIC [40]. In both cases, the EMPRO score reached 50 points because the alternative administration method was compared extensively with the original, but without assessing the whole range of metric properties. EPIC short forms were well rated (70 points), as good metric properties were demonstrated for both EPIC-26 and EPIC-Clinical Practice, as well as their comparability with scores of the original instrument. UCLA-PCI short form was rated low because only internal consistency reliability was estimated.

**Table 4:** Alternative forms of administration

Attribute	Administration forms		Short forms		
	EPIC—interactive voice response	UCLA-PCI-web-based mode	EPIC-26	EPIC-clinical practice	UCLA-PCI short form
Alternative forms	50	50	66.7	66.7	16.7
35. Metric characteristics of alternative forms	++	–	+++	+++	++
36. Comparability of alternative forms	+++	++++	+++	+++	–

Explanation: ++++ 4 (strongly agree), +++ 3, ++ 2, + 1 (strongly disagree), – no information. The higher the agreement the better the rating

## Discussion

In this study, we assessed the performance of patient self-reported HRQL instruments applicable for early-stage prostate cancer disease. Information regarding development process, metric properties, and administrative issues was obtained in systematic reviews of the literature and was evaluated by experts using a standardized tool. Of the eight instruments, the best rate according to EMPRO standard criteria was found for EPIC. Results obtained by UCLA-PCI, PORPUS and PC-QoL also support good performance, and therefore, their use should be recommended. FACT-P and PCSI scored slightly above the threshold of acceptable results, while ESCAP-CDV is far from this minimum quality criterion.

### EPIC and UCLA-PCI

The EPIC and UCLA-PCI scored the highest in the overall EMPRO assessment. In our study, both instruments were the best in “concept and measurement model,” and obtained very high “validity,” “responsiveness,” and “interpretability” results, where they were placed at second position. Despite these good results of UCLA-PCI, we recommend EPIC (its upgrade) not only due to its good reliability, but also because it incorporates a hormonal domain and urinary subscales for incontinence and irritative–obstructive symptoms (while UCLA-PCI’s urinary domain mainly queries incontinence). Both questionnaires have developed brief versions to minimize administration burden. The EPIC-26 [41] shortened to 10 min the time required to complete, and the EPIC for Clinical Practice [42] with 16 items was designed to be administered and scored directly during the clinical visit. The short UCLA-PCI [43] contains 14 of the original 20 items.

### PORPUS

PORPUS obtained the third best rating in the overall summary score. It is the only prostate cancer-specific instrument combining econometric and psychometric methods. As a result, it can be used as a preference-based health index obtaining utilities (PORPUS-U) for economic evaluation or as a short descriptive HRQL profile (PORPUS-P) [34]. In our metric quality evaluation, it was at the top for “validity” (maximum score), and it ranked second, equal to EPIC and UCLA-PCI, for “responsiveness” and “interpretability.” However, it just passed the requirements of “conceptual and measurement model” as experts highlighted the need to clarify the different elicitation methods to obtain utilities with PORPUS-U: direct methods with standard gamble or rating scale (PORPUS-U_SG_ and PORPUS-U_RS_), and an indirect method with standard gamble (PORPUS-U_I_) [44, 45]. EMPRO scores for reliability were low because the intraclass correlation coefficient of PORPUS-U was 0.66 [44] (lower than 0.7), and the test–retest design was insufficiently described. The PORPUS is the only prostate cancer-specific instrument for which general population-based norms exist to facilitate its score interpretation [46].

### PC-QoL and PCSI

The PC-QoL obtained the fourth best rating in the overall summary score. Despite being at the top on “reliability” and “responsiveness” and the second on “validity,” it is penalized for lacking information on “interpretability.” The first version [36] consisted of 52 items summarized in 10 domains. Befort et al. [47] revised the instrument and made it a 46-item questionnaire with eight scales that also provides adequate metric properties. The PCSI ranked sixth on the overall score and met the minimum quality criteria for all the attributes except “reliability.” The authors proposed the use of internal anchors employing the instrument’s distress or bother items to establish cutoff points (good, intermediate or poor function) [48]. This strategy was later deployed for the interpretation of other instruments such as EPIC and UCLA-PCI [49, 50]. It is the only instrument that considers patients’ cancer worry.

### FACT-P and EORTC QLQ-PR25

Overall performance of FACT-P was acceptable, while EORTC QLQ-PR25 did not reach the threshold of 50 points. FACT-P was at the top for “interpretability,” with a 2–3 point clinically meaningful change estimation using anchor-based and distribution-based methods [51], but it presented low scores on reliability mainly because of poor rates on study methods and internal consistency results (Cronbach’s *α* below 0.7 [33]). On the other hand, since the clinically meaningful change was estimated among patients suffering from metastatic hormone-refractory prostate cancer, its applicability for localized disease merits further research. EORTC QLQ-PR25 is strongly penalized due to the lack of information regarding its interpretability and for providing inadequate results on responsiveness. Experts highlighted that the coefficient used to estimate the magnitude of change was insufficiently described [32], and no comparison with a stable group had been performed. However, it should be taken into account that EORTC QLQ-PR25 was the newest instrument, and to date, it has few publications in biomedical literature databases. EORTC and FACT developed their modules simultaneously in several languages, which represent an advantage to consider when choosing an instrument for multicentric international studies requiring different country versions.

### Comparison with other evaluative reviews

Our work has both similarities and differences when compared to the three evaluative reviews [8, 21, 22]. Consistently with our findings, EPIC and UCLA-PCI are always among the most highly recommended [8, 21, 22]; PC-QoL [8, 21] and PORPUS [21] also obtained high ratings in other reviews; and the PCSI also met the minimum standard criteria to be recommended in the only other review where it was included [8]. On the other hand, the only major difference detected with respect to previous reviews concerns the recommendation of FACT-P module. Rnic et al. [8], similarly to our study, assigned it an unfavorable reliability evaluation according to the Cronbach’s *α* coefficient of 0.65 and 0.69 reported by Esper et al. [33]. Yet Hamoen et al. [21] and the Oxford group [22] recommended the FACT-P: the first article assigned full points to internal consistency [21], and the second one rated it with “some limited evidence in favor” [22]. These results suggest a higher exigency on the EMPRO requirements in comparison with other evaluations and differences on the evaluation criteria applied. Rnic et al. [8] examined only 4 criteria (comprehensiveness, subjectivity of experience, internal consistency and extent of validation), while the attributes considered in the other two evaluations [21, 22] are similar to the EMPRO content. However, the only tool that generates attribute scores which are based on multiple items (ranging from 2 to 7) is EMPRO, thus resulting in a more exhaustive and comprehensive evaluation.

### Study limitations

Our findings should be interpreted taking into account the study limitations. Firstly, the basis of our results is the information retrieved in systematic literature reviews conducted only in the PubMed database. Although it is the leading database in health sciences, we may have failed to identify all the published articles with information on development process, metric properties or administration issues. However, the sensitive search strategy specifically designed for each instrument, the additional hand search of references, as well as the double independent review process followed, may have minimized this problem. Secondly, the EMPRO evaluation is based on the quantity and quality of published evidence. A lack of evidence for a few EMPRO items or attributes penalizes the EMPRO scores, because the scoring algorithm counts any missing information as the worst possible rating. Nevertheless, to avoid a strong penalization, the EMPRO score is not calculated if more than half of the information is missing. Not presenting proposals for interpretability penalized the overall score for some of the instruments. Therefore, developing strategies to facilitate the interpretation of scores (such as estimating the minimal important difference by using anchor-based or distribution-based strategies, or providing reference values) is recommended. These interpretation proposals may help to extend these PRO measures beyond the research setting. Thirdly, EMPRO ratings may be biased by the individual expertise of the evaluators, although the double and independent review conducted, as well as a comprehensive description of each item, may have attenuated this concern. Fourthly, studies on metric properties from different country versions (EORTC PR25, EPIC, FACT-P and UCLA-PCI) were considered in our EMPRO evaluation. Although these country versions can add noise in one sense, they also provide valuable information about the generalizability of the psychometric data to these measures. Fifthly, although clinical trials can provide evidence on some metric properties such as validity, sensitivity to change or interpretability, none was included in our study. These trials were considered inappropriate because they were not specifically designed for the assessment of metric properties, nor included it as a secondary objective. For example, neither differences nor a lack of differences in PRO scores between trial arms could be interpreted as the instrument’s responsiveness if there is no clear underlying hypothesis about change. Finally, as the standard error of measurement was not considered separately in EMPRO, the only information on the precision of the inferences at the individual level is based on the reliability of the instrument. Therefore, we cannot address the usefulness of these eight instruments at the individual patient’s level.

## Conclusions

In conclusion, the evidence would currently support a preference for the use of EPIC, PORPUS and PC-QoL. Choosing among them will mainly depend on particular study requirements. For longitudinal studies or clinical trials, where responsiveness and reproducibility are the maximum priority, PC-QoL or EPIC would be recommended. For economic evaluations, PORPUS would be chosen as it allows cost-utility analysis. The brief versions might be preferred to minimize administration burden: EPIC short [41], EPIC-Clinical Practice [42] or short UCLA-PCI [43]. Our results facilitate the decision process regarding the correct instrument selection and its use and interpretation for a certain study purpose or setting.

## Electronic supplementary material

Below is the link to the electronic supplementary material.

Supplementary material 1 (PDF 15 kb)

Supplementary material 2 (PDF 50 kb)

## Footnotes

1
http://groups.eortc.be/qol/eortc-modules.

2
http://www.facit.org/FACITOrg/Questionnaires.

3
http://www.bibliopro.org/sobre_empro/index.html.

## References

[1] Ferlay J, Steliarova-Foucher E, Lortet-Tieulent J, Rosso S, Coebergh JW, Comber H (2013). Cancer incidence and mortality patterns in Europe: Estimates for 40 countries in 2012. European Journal of Cancer.

[2] Sanda MG, Dunn RL, Michalski J, Sandler HM, Northouse L, Hembroff L (2008). Quality of life and satisfaction with outcome among prostate-cancer survivors. New England Journal of Medicine.

[3] Miller DC, Sanda MG, Dunn RL, Montie JE, Pimentel H, Sandler HM (2005). Long-term outcomes among localized prostate cancer survivors: Health-related quality-of-life changes after radical prostatectomy, external radiation, and brachytherapy. Journal of Clinical Oncology.

[4] Chou R, Croswell JM, Dana T, Bougatsos C, Blazina I, Fu R (2011). Screening for prostate cancer: A review of the evidence for the U.S. Preventive Services Task Force. Annals of Internal Medicine.

[5] Calvert M, Blazeby J, Altman DG, Revicki DA, Moher D, Brundage MD (2013). Reporting of patient-reported outcomes in randomized trials: The CONSORT PRO extension. Journal of the American Medical Association.

[6] Osoba D, Tannock IF, Ernst DS, Neville AJ (1999). Health-related quality of life in men with metastatic prostate cancer treated with prednisone alone or mitoxantrone and prednisone. Journal of Clinical Oncology.

[7] Stockler MR, Osoba D, Goodwin P, Corey P, Tannock IF (1998). Responsiveness to change in health-related quality of life in a randomized clinical trial: A comparison of the Prostate Cancer Specific Quality of Life Instrument (PROSQOLI) with analogous scales from the EORTC QLQ-C30 and a trial specific module. European Organization for Research and Treatment of Cancer. Journal of Clinical Epidemiology.

[8] Rnic K, Linden W, Tudor I, Pullmer R, Vodermaier A (2013). Measuring symptoms in localized prostate cancer: A systematic review of assessment instruments. Prostate Cancer and Prostatic Diseases.

[9] Badia X, Baro E (2001). Cuestionarios de salud en España y su uso en atención primaria. Atencion Primaria.

[10] Mokkink LB, Terwee CB, Patrick DL, Alonso J, Stratford PW, Knol DL (2010). The COSMIN checklist for assessing the methodological quality of studies on measurement properties of health status measurement instruments: An international Delphi study. Quality of Life Research.

[11] Valderas JM, Ferrer M, Mendivil J, Garin O, Rajmil L, Herdman M (2008). Development of EMPRO: A tool for the standardized assessment of patient-reported outcome measures. Value Health.

[12] Scientific Advisory Committee of the Medical Outcomes Trust (2002). Assessing health status and quality-of-life instruments: Attributes and review criteria. Quality of Life Research.

[13] Garin, O., Herdman, M., Vilagut, G., Ferrer, M., Ribera, A., Rajmil, L., et al. (2014). Assessing health-related quality of life in heart failure: a systematic, standardized comparison of available measures. *Heart Failure Review*. doi:10.1007/s10741-013-9394-7. 10.1007/s10741-013-9394-7 23681849

[14] Schmidt, S., Ferrer, M., Gonzalez, M., Gonzalez, N., Valderas, J. M., Alonso, J., et al. (2014). Evaluation of shoulder-specific patient-reported outcome measures: A systematic and standardized comparison of available evidence. *Journal of Shoulder and Elbow Surgery,**23*, 434–444. 10.1016/j.jse.2013.09.029 24406123

[15] Efficace F, Bottomley A, van Andel G (2003). Health related quality of life in prostate carcinoma patients: A systematic review of randomized controlled trials. Cancer.

[16] Albaugh J, Hacker ED (2008). Measurement of quality of life in men with prostate cancer. Clinical Journal of Oncology Nursing.

[17] Namiki S, Arai Y (2010). Health-related quality of life in men with localized prostate cancer. International Journal of Urology.

[18] Quek ML, Penson DF (2005). Quality of life in patients with localized prostate cancer. Urologic Oncology.

[19] Sommers SD, Ramsey SD (1999). A review of quality-of-life evaluations in prostate cancer. Pharmacoeconomics.

[20] Penson DF (2007). Quality of life after therapy for localized prostate cancer. Cancer Journal.

[21] Hamoen, E. H., De Rooij, M., Witjes, J. A., Barentsz, J. O., & Rovers, M. M. (2014). Measuring health-related quality of life in men with prostate cancer: A systematic review of the most used questionnaires and their validity. *Urologic Oncology*. doi:10.1016/j.urolonc.2013.10.005. 10.1016/j.urolonc.2013.10.005 24433753

[22] Morris, C., Gibbons, E., Fitzpatrick, R. (2009). *A structured review of patient*-*reported outcome measures for men with prostate cancer*. University of Oxford. http://phi.uhce.ox.ac.uk/pdf/CancerReviews/PROMs_Oxford_Prostate%20Cancer_012011.pdf. Accessed March 24, 2013.

[23] Emery MP, Perrier LL, Acquadro C (2005). Patient-reported outcome and quality of life instruments database (PROQOLID): Frequently asked questions. Health and Quality of Life Outcomes.

[24] Cappelleri JC, Rosen RC (2005). The sexual health inventory for men (SHIM): A 5-year review of research and clinical experience. International Journal of Impotence Research.

[25] Fowler FJ, Barry MJ, Lu-Yao G, Roman A, Wasson J, Wennberg JE (1993). Patient-reported complications and follow-up treatment after radical prostatectomy. The national medicare experience: 1988–1990 (updated June 1993). Urology.

[26] Karakiewicz PI, Kattan MW, Tanguay S, Elhilali MM, Bazinet M, Scardino PT (2003). Cross-cultural validation of the UCLA prostate cancer index. Urology.

[27] Korfage IJ, Essink-Bot ML, Madalinska JB, Kirkels WJ, Litwin MS, de Koning HJ (2003). Measuring disease specific quality of life in localized prostate cancer: The Dutch experience. Quality of Life Research.

[28] Gacci M, Livi L, Paiar F, Detti B, Litwin MS, Bartoletti R (2005). Quality of life after radical treatment of prostate cancer: Validation of the Italian version of the University of California-Los Angeles Prostate Cancer Index. Urology.

[29] Kakehi Y, Kamoto T, Ogawa O, Arai Y, Litwin MS, Suzukamo Y (2002). Development of Japanese version of the UCLA Prostate Cancer Index: A pilot validation study. International Journal of Clinical Oncology.

[30] Krongrad A, Perczek RE, Burke MA, Granville LJ, Lai H, Lai S (1997). Reliability of Spanish translations of select urological quality of life instruments. Journal of Urology.

[31] Morales LA, Grau FG, Campoy MP, Benavente RA, del Pascual del Pobil Moreno JL (2002). Development of the ESCAP-CDV as measuring tool for the assessment of quality of life in prostatic cancer. Actas Urologicas Espanolas.

[32] van Andel G, Bottomley A, Fossa SD, Efficace F, Coens C, Guerif S (2008). An international field study of the EORTC QLQ-PR25: A questionnaire for assessing the health-related quality of life of patients with prostate cancer. European Journal of Cancer.

[33] Esper P, Mo F, Chodak G, Sinner M, Cella D, Pienta KJ (1997). Measuring quality of life in men with prostate cancer using the functional assessment of cancer therapy-prostate instrument. Urology.

[34] Krahn M, Ritvo P, Irvine J, Tomlinson G, Bezjak A, Trachtenberg J (2000). Construction of the Patient-Oriented Prostate Utility Scale (PORPUS): A multiattribute health state classification system for prostate cancer. Journal of Clinical Epidemiology.

[35] Wei JT, Dunn RL, Litwin MS, Sandler HM, Sanda MG (2000). Development and validation of the expanded prostate cancer index composite (EPIC) for comprehensive assessment of health-related quality of life in men with prostate cancer. Urology.

[36] Giesler RB, Miles BJ, Cowen ME, Kattan MW (2000). Assessing quality of life in men with clinically localized prostate cancer: Development of a new instrument for use in multiple settings. Quality of Life Research.

[37] Clark JA, Talcott JA (2001). Symptom indexes to assess outcomes of treatment for early prostate cancer. Medical Care.

[38] Litwin MS, Hays RD, Fink A, Ganz PA, Leake B, Brook RH (1998). The UCLA Prostate Cancer Index: Development, reliability, and validity of a health-related quality of life measure. Medical Care.

[39] Broering, J. M., Paciorek, A., Carroll, P. R., Wilson, L. S., Litwin, M. S., & Miaskowski, C. (2014). Measurement equivalence using a mixed-mode approach to administer health-related quality of life instruments. *Quality of Life Research,**23*, 495–508. 10.1007/s11136-013-0493-7 23943258

[40] Skolarus TA, Holmes-Rovner M, Hawley ST, Dunn RL, Barr KL, Willard NR (2012). Monitoring quality of life among prostate cancer survivors: The feasibility of automated telephone assessment. Urology.

[41] Szymanski KM, Wei JT, Dunn RL, Sanda MG (2010). Development and validation of an abbreviated version of the expanded prostate cancer index composite instrument for measuring health-related quality of life among prostate cancer survivors. Urology.

[42] Chang P, Szymanski KM, Dunn RL, Chipman JJ, Litwin MS, Nguyen PL (2011). Expanded prostate cancer index composite for clinical practice: Development and validation of a practical health related quality of life instrument for use in the routine clinical care of patients with prostate cancer. Journal of Urology.

[43] Litwin MS, McGuigan KA (1999). Accuracy of recall in health-related quality-of-life assessment among men treated for prostate cancer. Journal of Clinical Oncology.

[44] Ritvo P, Irvine J, Naglie G, Tomlinson G, Bezjak A, Matthew A (2005). Reliability and validity of the PORPUS, a combined psychometric and utility-based quality-of-life instrument for prostate cancer. Journal of Clinical Epidemiology.

[45] Tomlinson G, Bremner KE, Ritvo P, Naglie G, Krahn MD (2012). Development and validation of a utility weighting function for the patient-oriented prostate utility scale (PORPUS). Medical Decision Making.

[46] Waldmann A, Rohde V, Bremner K, Krahn M, Kuechler T, Katalinic A (2009). Measuring prostate-specific quality of life in prostate cancer patients scheduled for radiotherapy or radical prostatectomy and reference men in Germany and Canada using the Patient Oriented Prostate Utility Scale-Psychometric (PORPUS-P). BMC Cancer.

[47] Befort CA, Zelefsky MJ, Scardino PT, Borrayo E, Giesler RB, Kattan MW (2005). A measure of health-related quality of life among patients with localized prostate cancer: Results from ongoing scale development. Clinical Prostate Cancer.

[48] Chen RC, Clark JA, Talcott JA (2009). Individualizing quality-of-life outcomes reporting: How localized prostate cancer treatments affect patients with different levels of baseline urinary, bowel, and sexual function. Journal of Clinical Oncology.

[49] Bergman J, Kwan L, Litwin MS (2010). Improving decisions for men with prostate cancer: Translational outcomes research. Journal of Urology.

[50] Pardo Y, Guedea F, Aguilo F, Fernandez P, Macias V, Marino A (2010). Quality-of-life impact of primary treatments for localized prostate cancer in patients without hormonal treatment. Journal of Clinical Oncology.

[51] Cella D, Nichol MB, Eton D, Nelson JB, Mulani P (2009). Estimating clinically meaningful changes for the functional assessment of cancer therapy—Prostate: Results from a clinical trial of patients with metastatic hormone-refractory prostate cancer. Value Health.

