# Supplementary material for: Assessing quality of life in patients with prostate cancer: a systematic and standardized comparison of available instruments
Source: Qual Life Res. 2014 Apr 19;23(8):2169–81. doi: 10.1007/s11136-014-0678-8 (PMC4155169; doi:10.1007/s11136-014-0678-8)
Supplement: Supplementary file 1 — Supplementary material 1 (PDF 15 kb) [file 11136_2014_678_MOESM1_ESM.pdf]

Online Appendix 1. Specific search terminology for each instrument that was used in the systematic literature reviews.

**Estudio sobre la Calidad de Vida en el Cáncer de Próstata – ESCAP-CDV**
(("urologic neoplasms"[MeSH Terms] OR ("urologic"[All Fields] AND "neoplasms"[All Fields]) OR "urologic neoplasms"[All Fields]) OR ("prostatic neoplasms"[MeSH Terms] OR ("prostatic"[All Fields] AND "neoplasms"[All Fields]) OR "prostatic neoplasms"[All Fields] OR ("prostate"[All Fields] AND "cancer"[All Fields]) OR "prostate cancer"[All Fields])) AND ("quality of life"[MeSH Terms] OR ("quality"[All Fields] AND "life"[All Fields]) OR "quality of life"[All Fields]) AND ((Estudio[All Fields] AND sobre[All Fields] AND la[All Fields] AND Calidad[All Fields] AND ("drug effects"[Subheading] OR ("drug"[All Fields] AND "effects"[All Fields]) OR "drug effects"[All Fields] OR "de"[All Fields]) AND Vida[All Fields] AND ("enzymology"[Subheading] OR "enzymology"[All Fields] OR "en"[All Fields]) AND el[All Fields] AND ("neoplasms"[MeSH Terms] OR "neoplasms"[All Fields] OR "cancer"[All Fields]) AND ("drug effects"[Subheading] OR ("drug"[All Fields] AND "effects"[All Fields]) OR "drug effects"[All Fields] OR "de"[All Fields]) AND ("prostate"[MeSH Terms] OR "prostate"[All Fields] OR "prostata"[All Fields])) OR "ESCAP-CDV"[All Fields])
**European Organisation for Research and Treatment of Cancer - Quality of Life - Prostate Cancer Module – EORTC-QLQ PR25**
(("urologic neoplasms"[MeSH Terms] OR ("urologic"[All Fields] AND "neoplasms"[All Fields]) OR "urologic neoplasms"[All Fields]) OR ("prostatic neoplasms"[MeSH Terms] OR ("prostatic"[All Fields] AND "neoplasms"[All Fields]) OR "prostatic neoplasms"[All Fields] OR ("prostate"[All Fields] AND "cancer"[All Fields]) OR "prostate cancer"[All Fields])) AND ("quality of life"[MeSH Terms] OR ("quality"[All Fields] AND "life"[All Fields]) OR "quality of life"[All Fields]) AND ("EORTC"[All Fields] OR "European Organisation for Research and Treatment of Cancer"[All Fields])
**Expanded Prostate Cancer Index Composite – EPIC**
(("urologic neoplasms"[MeSH Terms] OR ("urologic"[All Fields] AND "neoplasms"[All Fields]) OR "urologic neoplasms"[All Fields]) OR ("prostatic neoplasms"[MeSH Terms] OR ("prostatic"[All Fields] AND "neoplasms"[All Fields]) OR "prostatic neoplasms"[All Fields] OR ("prostate"[All Fields] AND "cancer"[All Fields]) OR "prostate cancer"[All Fields])) AND (EPIC[All Fields] OR (expanded[All Fields] AND ("prostatic neoplasms"[MeSH Terms] OR ("prostatic"[All Fields] AND "neoplasms"[All Fields]) OR "prostatic neoplasms"[All Fields] OR ("prostate"[All Fields] AND "cancer"[All Fields]) OR "prostate cancer"[All Fields]) AND ("abstracting and indexing as topic"[MeSH Terms] OR ("abstracting"[All Fields] AND "indexing"[All Fields] AND "topic"[All Fields]) OR "abstracting and indexing as topic"[All Fields] OR "index"[All Fields]) AND composite[All Fields]))
**Functional Assessment of Cancer Therapy - Prostate Cancer Module – FACT-P**
(("urologic neoplasms"[MeSH Terms] OR ("urologic"[All Fields] AND "neoplasms"[All Fields]) OR "urologic neoplasms"[All Fields]) OR ("prostatic neoplasms"[MeSH Terms] OR ("prostatic"[All Fields] AND "neoplasms"[All Fields]) OR "prostatic neoplasms"[All Fields] OR ("prostate"[All Fields] AND "cancer"[All Fields]) OR "prostate cancer"[All Fields])) AND ("quality of life"[MeSH Terms] OR ("quality"[All Fields] AND "life"[All Fields]) OR "quality of life"[All Fields]) AND ("FACT"[All Fields] OR "functional assessment of cancer therapy"[All Fields])

**Patient-Oriented Prostate Utility Scale – PORPUS**
(("urologic neoplasms"[MeSH Terms] OR ("urologic"[All Fields] AND "neoplasms"[All Fields]) OR "urologic neoplasms"[All Fields]) OR ("prostatic neoplasms"[MeSH Terms] OR ("prostatic"[All Fields] AND "neoplasms"[All Fields]) OR "prostatic neoplasms"[All Fields] OR ("prostate"[All Fields] AND "cancer"[All Fields]) OR "prostate cancer"[All Fields])) AND (PORPUS[All Fields] OR "patient-oriented prostate utility scale"[All Fields])
**Prostate Cancer Quality of Life Instrument – PC-QoL**
(("urologic neoplasms"[MeSH Terms] OR ("urologic"[All Fields] AND "neoplasms"[All Fields]) OR "urologic neoplasms"[All Fields]) OR ("prostatic neoplasms"[MeSH Terms] OR ("prostatic"[All Fields] AND "neoplasms"[All Fields]) OR "prostatic neoplasms"[All Fields] OR ("prostate"[All Fields] AND "cancer"[All Fields]) OR "prostate cancer"[All Fields])) AND ("quality of life"[MeSH Terms] OR ("quality"[All Fields] AND "life"[All Fields]) OR "quality of life"[All Fields]) AND (PC-QoL[All Fields] OR PCQoL[All Fields] OR (("prostatic neoplasms"[MeSH Terms] OR ("prostatic"[All Fields] AND "neoplasms"[All Fields]) OR "prostatic neoplasms"[All Fields] OR ("prostate"[All Fields] AND "cancer"[All Fields]) OR "prostate cancer"[All Fields]) AND ("quality of life"[MeSH Terms] OR ("quality"[All Fields] AND "life"[All Fields]) OR "quality of life"[All Fields]) AND instrument[All Fields]))
**Prostate Cancer Symptom Indices – PCSI**
(("urologic neoplasms"[MeSH Terms] OR ("urologic"[All Fields] AND "neoplasms"[All Fields]) OR "urologic neoplasms"[All Fields]) OR ("prostatic neoplasms"[MeSH Terms] OR ("prostatic"[All Fields] AND "neoplasms"[All Fields]) OR "prostatic neoplasms"[All Fields] OR ("prostate"[All Fields] AND "cancer"[All Fields]) OR "prostate cancer"[All Fields])) AND ("quality of life"[MeSH Terms] OR ("quality"[All Fields] AND "life"[All Fields]) OR "quality of life"[All Fields]) AND (TALCOTT[All Fields] OR (TALCOTT[All Fields] AND instrument[All Fields]) OR (TALCOTT[All Fields] AND ("weights and measures"[MeSH Terms] OR ("weights"[All Fields] AND "measures"[All Fields]) OR "weights and measures"[All Fields] OR "scale"[All Fields])) OR (TALCOTT[All Fields] AND ("questionnaires"[MeSH Terms] OR "questionnaires"[All Fields] OR "questionnaire"[All Fields])))
**University of California, Los Angeles - Prostate Cancer Index – UCLA-PCI**
(("urologic neoplasms"[MeSH Terms] OR ("urologic"[All Fields] AND "neoplasms"[All Fields]) OR "urologic neoplasms"[All Fields]) OR ("prostatic neoplasms"[MeSH Terms] OR ("prostatic"[All Fields] AND "neoplasms"[All Fields]) OR "prostatic neoplasms"[All Fields] OR ("prostate"[All Fields] AND "cancer"[All Fields]) OR "prostate cancer"[All Fields])) AND ("quality of life"[MeSH Terms] OR ("quality"[All Fields] AND "life"[All Fields]) OR "quality of life"[All Fields]) AND (UCLA-PCI[All Fields] OR (("universities"[MeSH Terms] OR "universities"[All Fields] OR "university"[All Fields]) AND ("california"[MeSH Terms] OR "california"[All Fields]) AND ("los angeles"[MeSH Terms] OR ("los"[All Fields] AND "angeles"[All Fields]) OR "los angeles"[All Fields]) AND ("prostatic neoplasms"[MeSH Terms] OR ("prostatic"[All Fields] AND "neoplasms"[All Fields]) OR "prostatic neoplasms"[All Fields] OR ("prostate"[All Fields] AND "cancer"[All Fields]) OR "prostate cancer"[All Fields]) AND ("abstracting and indexing as topic"[MeSH Terms] OR ("abstracting"[All Fields] AND "indexing"[All Fields] AND "topic"[All Fields]) OR "abstracting and indexing as topic"[All Fields] OR "index"[All Fields])) OR "prostate cancer index"[All Fields])
