# Supplementary material for: Assessing quality of life in patients with prostate cancer: a systematic and standardized comparison of available instruments
Source: Qual Life Res. 2014 Apr 19;23(8):2169–81. doi: 10.1007/s11136-014-0678-8 (PMC4155169; doi:10.1007/s11136-014-0678-8)
Supplement: Supplementary file 2 — Supplementary material 2 (PDF 50 kb) [file 11136_2014_678_MOESM2_ESM.pdf]

Online Appendix 2. Bibliographic references retrieved in the systematic literature review and identified to be included in the EMPRO expert evaluation.

**Estudio sobre la Calidad de Vida en el Cáncer de Próstata – ESCAP-CDV**
(1) Morales López A, Grau Fibla G, Campoy Martínez P, Benavente Rodríguez A, Pascual del Pobil Moreno JL. [Development of the ESCAP-CDV as measuring tool for the assessment of quality of life in prostatic cancer]. Actas Urol Esp. 2002 Apr;26(4):242-9.
(2) Morales López A, Grau Fibla G, Campoy Martínez P, Benavente Rodríguez A, Pascual del Pobil Moreno JL. [Validation of ESCAP-CD as an instrument of measure for the evaluation of the quality of life in prostatic cancer. Part 2a]. Actas Urol Esp. 2002 May;26(5):320-34.
**European Organisation for Research and Treatment of Cancer - Quality of Life - Prostate Cancer Module – EORTC-QLQ PR25**
(1) Chang YJ, Liang WM, Wu HC, Lin HC, Wang JY, Li TC, Yeh YC, Chang CH. Psychometric evaluation of the Taiwan Chinese version of the EORTC QLQ-PR25 for HRQOL assessment in prostate cancer patients. Health Qual Life Outcomes 2012; 10:96.
(2) Arraras JI, Villafranca E, Arias dl, V, Romero P, Rico M, Vila M et al. The EORTC Quality of Life Questionnaire for patients with prostate cancer: EORTC QLQ-PR25. Validation study for Spanish patients. Clin Transl Oncol 2009; 11(3):160-164.
(3) van Andel G, Bottomley A, Fossa SD, Efficace F, Coens C, Guerif S et al. An international field study of the EORTC QLQ-PR25: a questionnaire for assessing the health-related quality of life of patients with prostate cancer. Eur J Cancer 2008; 44(16):2418-2424.
(4) Wadasadawala T, Murthy V, Mahantshetty U, Engineer R, Shrivastava S, Dinshaw K. The European Organization for Research and Treatment of Cancer prostate-specific quality of life module (PR-25) in Hindi and Marathi: translation and pilot testing process. J Cancer Res Ther 2008; 4(2):64-69.
(5) Borghede G, Sullivan M. Measurement of quality of life in localized prostatic cancer patients treated with radiotherapy. Development of a prostate cancer-specific module supplementing the EORTC QLQ-C30. Qual Life Res 1996; 5(2):212-22.
**Expanded Prostate Cancer Index Composite – EPIC**
(1) Alves E, Medina R, Andreoni C. Validation of the Brazilian version of the Expanded Prostate Cancer Index Composite (EPIC) for patients submitted to radical prostatectomy. Int Braz J Urol 2013; 39(3):344-352.
(2) Skolarus TA, Holmes-Rovner M, Hawley ST, Dunn RL, Barr KL, Willard NR, Wei JT, Piette JD, An LC. Monitoring quality of life among prostate cancer survivors: the feasibility of automated telephone assessment. Urology 2012; 80(5):1021-1026.
(3) Rodrigues G, Bauman G, Venkatesan V, Ahmad B, Lock M, Sexton T, D'Souza D, Stitt L, Eid S. Cross validation of the prostate cancer radiotherapy late toxicity (PCRT) questionnaire with the expanded prostate cancer index composite (EPIC) instrument. Can J Urol 2011; 18(4):5802.
(4) Chung KJ, Kim JJ, Lim SH, Kim TH, Han DH, Lee SW. Development and validation of the korean version of expanded prostate cancer index composite: questionnaire assessing health-related quality of life after prostate cancer treatment. Korean J Urol 2010; 51(9):601-612.

(5) Levinson AW, Ward NT, Sanda MG, Mettee LZ, Wei JT, Su LM, Litwin MS, Pavlovich CP. Comparison of validated instruments measuring sexual function in men. Urology. 2010 Aug;76(2):380-6.
(6) Pardo Y, Guedea F, Aguiló F, Fernández P, Macías V, Mariño A, Hervás A, Herruzo I, Ortiz MJ, Ponce de León J, Craven-Bratle J, Suárez JF, Boladeras A, Pont À, Ayala A, Sancho G, Martínez E, Alonso J, Ferrer M. Quality-of life impact of primary treatments for localized prostate cancer in patients without hormonal treatment. J Clin Oncol. 2010 Nov 1;28(31):4687-96.
(7) Ferrer M, Garin O, Pera J, Prats JM, Mendivil J, Alonso J et al. [Evaluation of the quality of life of patients with localizad prostate cancer: validation of the Spanish version of the EPIC]. Med Clin (Barc) 2009; 132(4):128
(8) Hedgepeth RC, Labo J, Zhang L, Wood DP, Jr. Expanded Prostate Cancer Index Composite versus Incontinence Symptom Index and Sexual Health Inventory for Men to measure functional outcomes after prostatectomy. J Urol 2009; 182(1):221-227.
(9) Schroeck FR, Donatucci CF, Smathers EC, Sun L, Albala DM, Polascik TJ et al. Defining potency: a comparison of the International Index of Erectile Function short version and the Expanded Prostate Cancer Index Composite. Cancer 2008; 113(10):2687-2694.
(10) Kakehi Y, Takegami M, Suzukamo Y, Namiki S, Arai Y, Kamoto T et al. Health related quality of life in Japanese men with localized prostate cancer treated with current multiple modalities assessed by a newly developed Japanese version of the Expanded Prostate Cancer Index Composite. J Urol 2007; 177(5):1856-1861.
(11) Namiki S, Takegami M, Kakehi Y, Suzukamo Y, Fukuhara S, Arai Y. Analysis linking UCLA PCI with Expanded Prostate Cancer Index Composite: an evaluation of health related quality of life in Japanese men with localized prostate cancer. J Urol 2007; 178(2):473-477.
(12) Muanza TM, Albert PS, Smith S, Godette D, Crouse NS, Cooley-Zgela T et al. Comparing measures of acute bowel toxicity in patients with prostate cancer treated with external beam radiation therapy. Int J Radiat Oncol Biol Phys 2005; 62(5):1316-1321.
(13) Wei JT, Dunn RL, Litwin MS, Sandler HM, Sanda MG. Development and validation of the expanded prostate cancer index composite (EPIC) for comprehensive assessment of health-related quality of life in men with prostate cancer. Urology 2000; 56(6):899-905.
**Functional Assessment of Cancer Therapy - Prostate Cancer Module – FACT-P**
(1) Chung KJ, Kim JJ, Lim SH, Kim TH, Han DH, Lee SW. Development and validation of the korean version of expanded prostate cancer index composite: questionnaire assessing health-related quality of life after prostate cancer treatment. Korean J Urol 2010; 51(9):601-612.
(2) Cella D, Nichol MB, Eton D, Nelson JB, Mulani P. Estimating clinically meaningful changes for the Functional Assessment of Cancer Therapy-Prostate: results from a clinical trial of patients with metastatic hormone-refractory prostate cancer. Value Health 2009; 12(1):124-129.
(3) Ferrer M, Garin O, Pera J, Prats JM, Mendivil J, Alonso J et al. [Evaluation of the quality of life of patients with localizad prostate cancer: validation of the Spanish version of the EPIC]. Med Clin (Barc) 2009; 132(4):128-135.

(4) Stone PC, Murphy RF, Matar HE, Almerie MQ. Quality of life in patients with prostate cancer: development and application of a hybrid assessment method. Prostate Cancer Prostatic Dis 2009; 12(1):72-77.

(5) Stone PC, Murphy RF, Matar HE, Almerie MQ. Measuring the individual quality of life of patients with prostate cancer. Prostate Cancer Prostatic Dis 2008; 11(4):390-396.

(6) Wu EQ, Mulani P, Farrell MH, Sleep D. Mapping FACT-P and EORTC QLQ-C30 to patient health status measured by EQ-5D in metastatic hormone-refractory prostate cancer patients. Value Health 2007; 10(5):408-414.

(7) Hong JH, Jeon SS, Lee HM, Choi YH, Kim S, Choi HY. The Functional Assessment of Cancer Therapy-Prostate (FACT-P) scales in men with prostate cancer: reliability and validity of the Korean version. J Korean Med Sci 2006; 21(2):295-299.

(8) Akaza H, Naito S, Chang SJ, Chen KK, Cheng C, Choi HY et al. The 3rd Conference on Asian Trends in Prostate Cancer Hormone Therapy. Gan To Kagaku Ryoho 2004; 31(8):1285-1295.

(9) Batista Miranda JE, Sevilla-Cecilia C, Torrubia R, Musquera M, Huguet-Pérez J, Ponce de Leon X, et al. Quality of life in prostate cáncer and controls: psychometric validation of the FACTP-4 Spanish, and relation to urinary symptoms. Arch Esp Urol. 2003;56:447-54.

(10) Yount S, Cella D, Banik D, Ashraf T, Shevrin D. Brief assessment of priority symptoms in hormone refractory prostate cancer: the FACT Advanced Prostate Symptom Index (FAPSI). Health Qual Life Outcomes 2003; 1:69.

(11) Wei JT, Dunn RL, Litwin MS, Sandler HM, Sanda MG. Development and validation of the expanded prostate cancer index composite (EPIC) for comprehensive assessment of health-related quality of life in men with prostate cancer. Urology 2000; 56(6):899-905.

(12) Esper P, Mo F, Chodak G, Sinner M, Cella D, Pienta KJ. Measuring quality of life in men with prostate cancer using the functional assessment of cancer therapy-prostate instrument. Urology 1997; 50(6):920-928.

Patient-Oriented Prostate Utility Scale – PORPUS

(1) Tomlinson G, Bremner KE, Ritvo P, Naglie G, Krahn MD. Development and validation of a utility weighting function for the patient-oriented prostate utility scale (PORPUS). Med Decis Making 2012; 32(1):11-30.

(2) Waldmann A, Rohde V, Bremner K, Krahn M, Kuechler T, Katalinic A. Measuring prostate-specific quality of life in prostate cancer patients scheduled for radiotherapy or radical prostatectomy and reference men in Germany and Canada using the Patient Oriented Prostate Utility Scale-Psychometric (PORPUS-P). BMC Cancer 2009; 9:295.

(3) Krahn M, Bremner KE, Tomlinson G, Ritvo P, Irvine J, Naglie G. Responsiveness of disease-specific and generic utility instruments in prostate cancer patients. Qual Life Res 2007; 16(3):509-522.

(4) Ritvo P, Irvine J, Naglie G, Tomlinson G, Bezjak A, Matthew A et al. Reliability and validity of the PORPUS, a combined psychometric and utility-based quality-of-life instrument for prostate cancer. J Clin Epidemiol 2005; 58(5):466-474.

(5) Krahn M, Ritvo P, Irvine J, Tomlinson G, Bezjak A, Trachtenberg J et al. Construction of the Patient-Oriented Prostate Utility Scale (PORPUS): a multiattribute health state classification system for prostate cancer. J Clin Epidemiol 2000; 53(9):920-930.

Prostate Cancer Quality of Life Instrument – PC-QoL

(1) Befort CA, Zelefsky MJ, Scardino PT, Borrayo E, Giesler RB, Kattan MW. A measure of health-related quality of life among patients with localized prostate cancer: results from ongoing scale development. Clin Prostate Cancer 2005; 4(2):100-108.

(2) Giesler RB, Given B, Given CW, Rawl S, Monahan P, Burns D et al. Improving the quality of life of patients with prostate carcinoma: a randomized trial testing the efficacy of a nurse-driven intervention. Cancer 2005; 104(4):752-762.

(3) Giesler RB, Miles BJ, Cowen ME, Kattan MW. Assessing quality of life in men with clinically localized prostate cancer: development of a new instrument for use in multiple settings. Qual Life Res 2000; 9(6):645-665.

Prostate Cancer Symptom Indices – PCSI

(1) Chen RC, Clark JA, Talcott JA. Individualizing quality-of-life outcomes reporting: how localized prostate cancer treatments affect patients with different levels of baseline urinary, bowel, and sexual function. J Clin Oncol 2009; 27(24):3916-22.

(2) Talcott JA, Clark JA, Manola J, Mitchell SP. Bringing prostate cancer quality of life research back to the bedside: translating numbers into a format that patients can understand. J Urol 2006; 176(4):1558-64.

(3) Clark JA, Bokhour BG, Inui TS, Silliman RA, Talcott JA. Measuring patients' perceptions of the outcomes of treatment for early prostate cancer. Med Care.2003;41(8):923-36.

(4) Clark JA, Talcott JA. Symptom indexes to assess outcomes of treatment for early prostate cancer. Med Care 2001; 39(10):1118-1130.

(5) Talcott JA, Rieker P, Clark JA, Propert KJ, Weeks JC, Beard CJ et al. Patient-reported symptoms after primary therapy for early prostate cancer: results of a prospective cohort study. J Clin Oncol 1998; 16(1):275-283.

University of California, Los Angeles - Prostate Cancer Index – UCLA-PCI

(1) Broering JM, Paciorek A, Carroll PR, Wilson LS, Litwin MS, Miaskowski C. Measurement equivalence using a mixed-mode approach to administer health-related quality of life instruments. Qual Life Res 2013.

(2) Bergman J, Kwan L, Litwin MS. Improving decisions for men with prostate cancer: translational outcomes research. J Urol 2010; 183(6):2186-2192.

(3) Bergman J, Saigal CS, Kwan L, Litwin MS. Responsiveness of the University of California-Los Angeles Prostate Cancer Index. Urology 2010; 75(6):1418-1423.

(4) Levinson AW, Ward NT, Sanda MG, Mettee LZ, Wei JT, Su LM, Litwin MS, Pavlovich CP. Comparison of validated instruments measuring sexual function in men. Urology. 2010 Aug;76(2):380-6.

(5) Krahn M, Bremner KE, Tomlinson G, Ritvo P, Irvine J, Naglie G. Responsiveness of disease-specific and generic utility instruments in prostate cancer patients. Qual Life Res 2007; 16(3):509-522.
(6) Namiki S, Takegami M, Kakehi Y, Suzukamo Y, Fukuhara S, Arai Y. Analysis linking UCLA PCI with Expanded Prostate Cancer Index Composite: an evaluation of health related quality of life in Japanese men with localized prostate cancer. J Urol 2007; 178(2):473-477.
(7) Gacci M, Livi L, Paiar F, Detti B, Litwin MS, Bartoletti R et al. Quality of life after radical treatment of prostate cancer: validation of the Italian version of the University of California-Los Angeles Prostate Cancer Index. Urology 2005; 66(2):338-343.
(8) Lebeau T, Perrotte P, Valiquette L, Benard F, McCormack M, Saad F et al. Validation of prostate cancer index and SF-12 short forms. Can J Urol 2005; 12(6):2873-2879.
(9) Karakiewicz PI, Kattan MW, Tanguay S, Elhilali MM, Bazinet M, Scardino PT et al. Cross-cultural validation of the UCLA prostate cancer index. Urology 2003; 61(2):302-307.
(10) Korfage IJ, Essink-Bot ML, Madalinska JB, Kirkels WJ, Litwin MS, de Koning HJ. Measuring disease specific quality of life in localized prostate cancer: the Dutch experience. Qual Life Res 2003; 12(4):459-464.
(11) Kakehi Y, Kamoto T, Ogawa O, Arai Y, Litwin MS, Suzukamo Y et al. Development of Japanese version of the UCLA Prostate Cancer Index: a pilot validation study. Int J Clin Oncol 2002; 7(5):306-311.
(12) Livsey JE, Routledge J, Burns M, Swindell R, Davidson SE, Cowan RA et al. Scoring of treatment-related late effects in prostate cancer. Radiother Oncol 2002; 65(2):109-121.
(13) Krongrad A, Litwin MS, Lai H, Lai S. Dimensions of quality of life in prostate cancer. J Urol 1998; 160(3 Pt 1):807-810.
(14) Litwin MS, Hays RD, Fink A, Ganz PA, Leake B, Brook RH. The UCLA Prostate Cancer Index: development, reliability, and validity of a health-related quality of life measure. Med Care 1998; 36(7):1002-1012.
(15) Krongrad A, Perczek RE, Burke MA, Granville LJ, Lai H, Lai S. Reliability of Spanish translations of select urological quality of life instruments. J Urol 1997; 158(2):493-496.
(16) Lubeck DP, Litwin MS, Henning JM, Carroll PR. Measurement of health-related quality of life in men with prostate cancer: the CaPSURE database. Qual Life Res 1997; 6(5):385-392.
